# Species identification, discovery, and biomonitoring: Strategic priorities for DNA barcoding in Europe, set in a global context

**DOI:** 10.1093/biosci/biag052

**Published:** 2026-05-22

**Authors:** Peter M Hollingsworth, Kasia Fantoni, Dimitris Koureas, Marine Arakelyan, Filippos A Aravanopoulos, Karolina Bacela-Spychalska, Liliana Ballesteros-Mejia, Pedro Beja, Georgi N Bonchev, Magda Bou Dagher Kharrat, Sarah J Bourlat, Tinatin Chkhartishvili, Fedor Čiampor, Filipe O Costa, Zoltán Csabai, Gabriela Dankova, Torbjørn Ekrem, Brent Emerson, Sónia Ferreira, Piotr Gadawski, Konstantinos Gkagkavouzis, Paul D N Hebert, Mihael C Ichim, Mišel Jelić, Elisavet Kaitetzidou, Belma Kalamujić Stroil, Stefaniya Kamenova, Emre Keskin, Alex Laini, Mara Lawniczak, Panagiotis Madesis, Matteo Montagna, Marko Mutanen, Ralph S Peters, Ben Price, Tomasz Rewicz, Rodolphe Rougerie, Björn Rulik, Nikolaus Szucsich, Rutger Vos, William P Goodall-Copestake, Alexandros Triantafyllidis, Michał Grabowski

**Affiliations:** Royal Botanic Garden Edinburgh, 20A Inverleith Row, Edinburgh EH3 5LR, United Kingdom; Naturalis Biodiversity Center, Darwinweg 2, 2333 CR Leiden, the Netherlands; Naturalis Biodiversity Center, Darwinweg 2, 2333 CR Leiden, the Netherlands; Faculty of Biology, Yerevan State University, Alek Manoogian 1, Yerevan 0025, Armenia; Faculty of Forestry and Natural Environment, Aristotle University of Thessaloniki, University Campus, PO Box 238, Thessaloniki 54124, Greece; Department of Invertebrate Zoology & Hydrobiology, Faculty of Biology & Environmental Protection, University of Lodz, Banacha 12/16, 90-237 Łódź, Poland; Direction de l’Expertise–DGD-REVE, Muséum national d’Histoire naturelle, Paris 75005, France; CIBIO, Centro de Investigação em Biodiversidade e Recursos Genéticos, InBIO Laboratório Associado, Campus de Vairão, Universidade do Porto, 4485-661 Vairão, Vila do Conde, Portugal; BIOPOLIS Program in Genomics, Biodiversity and Land Planning, CIBIO, Campus de Vairão, 4485-661 Vairão, Portugal; Institute of Plant Physiology and Genetics, Bulgarian Academy of Sciences, Acad. Georgi Bonchev St., Bldg. 21, 1113 Sofia, Bulgaria; European Forest Institute, Carrer Sant Antoni M. Claret, 167, 08025 Barcelona, Spain; Leibniz Institute for the Analysis of Biodiversity Change, Museum Koenig Bonn, Adenauerallee 160, 53113 Bonn, Germany; Institute of Zoology, Ilia State University, Cholokashvili av. 3/5, Tbilisi 0162, Georgia; Plant Science and Biodiversity Centre, Slovak Academy of Sciences, Dúbravská cesta 9, 84523 Bratislava, Slovakia; Centre of Molecular and Environmental Biology (CBMA) and ARNET–Aquatic Research Network, Department of Biology, University of Minho, Campus de Gualtar, 4710-057 Braga, Portugal; Institute of Science and Innnovation for Bio-Sustainability (IB-S), University of Minho, 4710-057 Braga, Portugal; Department of Hydrobiology, University of Pécs, Ifjúság útja 6, Pécs 7624, Hungary; HUN-REN Balaton Limnological Research Institute, Klebelsberg Kunó 3, Tihany 8237, Hungary; Naturalis Biodiversity Center, Darwinweg 2, 2333 CR Leiden, the Netherlands; Department of Natural History, NTNU University Museum, Norwegian University of Science and Technology, NO-7491 Trondheim, Norway; Island Ecology and Evolution Research Group, Institute of Natural Products and Agrobiology (IPNA-CSIC), San Cristóbal de La Laguna, Santa Cruz de Tenerife, Spain; CIBIO, Centro de Investigação em Biodiversidade e Recursos Genéticos, InBIO Laboratório Associado, Campus de Vairão, Universidade do Porto, 4485-661 Vairão, Vila do Conde, Portugal; BIOPOLIS Program in Genomics, Biodiversity and Land Planning, CIBIO, Campus de Vairão, 4485-661 Vairão, Portugal; Department of Invertebrate Zoology & Hydrobiology, Faculty of Biology & Environmental Protection, University of Lodz, Banacha 12/16, 90-237 Łódź, Poland; Department of Genetics, Development and Molecular Biology, School of Biology, Aristotle University of Thessaloniki, University Campus, Thessaloniki 54124, Greece; Genomics and Epigenomics Translational Research (GENeTres) Center for Interdisciplinary Research and Innovation (CIRI-AUTH), CIRI AUTh (KEDEK), Balkan Center, Buildings A and B, 10th km, Thessalonikis-Thermis 57001, Greece; Centre for Biodiversity Genomics, University of Guelph, Guelph, ON, N1G 2W1, Canada; National Institute of Research and Development for Biological Sciences, “Stejarul” Research Centre for Biological Sciences, Alexandru cel Bun St., 6, Piatra Neamt 610004, Romania; Department of Natural Sciences, Varaždin City Museum, Šetalište Josipa Jurja Strossmayera 3, 42000 Varaždin, Croatia; Department of Genetics, Development and Molecular Biology, School of Biology, Aristotle University of Thessaloniki, University Campus, Thessaloniki 54124, Greece; Genomics and Epigenomics Translational Research (GENeTres) Center for Interdisciplinary Research and Innovation (CIRI-AUTH), CIRI AUTh (KEDEK), Balkan Center, Buildings A and B, 10th km, Thessalonikis-Thermis 57001, Greece; University of Sarajevo-Institute for Genetic Engineering and Biotechnology, Zmaja od Bosne 8, 71000 Sarajevo, Bosnia and Herzegovina; National Museum of Natural History, Bulgarian Academy of Sciences, 1 Tsar Osvoboditel Blvd, 1000 Sofia, Bulgaria; Department of Fisheries and Aquaculture, Faculty of Agriculture, Evolutionary Genetics Laboratory (eGL), Ankara University, Subayevleri, 06135 Keçiören/Ankara, Türkiye; AgriGenomics Hub (AgriGx), Animal and Plant Genomics Research Innovation Centre, Kalaba, Kütükçü Alibey Cd. No: 2A, 06135 Keçiören/Ankara, Türkiye; Biodiversity Research Association (BAD), Kalaba, Kütükçü Alibey Cd. No: 2A, 06135 Keçiören/Ankara, Türkiye; Department of Life Sciences and Systems Biology, University of Torino, via Accademia Albertina 13, 10123 Torino, Italy; Wellcome Sanger Institute, Wellcome Genome Campus, Hinxton CB10 1SA, United Kingdom; Laboratory of Molecular Biology of Plants, School of Agricultural Sciences, University of Thessaly, 38221 Volos, Greece; Institute of Applied Biosciences, Centre for Research and Technology Hellas, 57001 Thessaloniki, Greece; Department of Agricultural Sciences, University of Naples Federico II, Piazza Carlo di Borbone 1, 80055 Portici, Italy; Ecology and Genetics Research Unit, University of Oulu, Oulu 90014, Finland; Leibniz Institute for the Analysis of Biodiversity Change, Museum Koenig Bonn, Adenauerallee 160, 53113 Bonn, Germany; Natural History Museum, Cromwell Road, London SW7 5BD, United Kingdom; Department of Invertebrate Zoology & Hydrobiology, Faculty of Biology & Environmental Protection, University of Lodz, Banacha 12/16, 90-237 Łódź, Poland; Institut de Systématique, Évolution, Biodiversité, UMR 7205 Muséum national d’Histoire naturelle, CNRS, Sorbonne Université, EPHE-PSL, Université des Antilles, Paris 75005, France; Leibniz Institute for the Analysis of Biodiversity Change, Museum Koenig Bonn, Adenauerallee 160, 53113 Bonn, Germany; Central Research Laboratories, National History Museum Vienna, Burgring 7, 1010 Vienna, Austria; Naturalis Biodiversity Center, Darwinweg 2, 2333 CR Leiden, the Netherlands; Royal Botanic Garden Edinburgh, 20A Inverleith Row, Edinburgh EH3 5LR, United Kingdom; Department of Genetics, Development and Molecular Biology, School of Biology, Aristotle University of Thessaloniki, University Campus, Thessaloniki 54124, Greece; Genomics and Epigenomics Translational Research (GENeTres) Center for Interdisciplinary Research and Innovation (CIRI-AUTH), CIRI AUTh (KEDEK), Balkan Center, Buildings A and B, 10th km, Thessalonikis-Thermis 57001, Greece; Department of Invertebrate Zoology & Hydrobiology, Faculty of Biology & Environmental Protection, University of Lodz, Banacha 12/16, 90-237 Łódź, Poland

**Keywords:** DNA barcoding, species identification, species discovery, biodiversity genomics, metabarcoding

## Abstract

The International Barcode of Life (iBOL) initiative is building a globally accessible DNA-based system for species identification and discovery. This paper outlines the mission and strategic priorities for the iBOL community in Europe (iBOL Europe), set in a global context. The mission of iBOL Europe is to produce, curate, and provide access to a complete DNA barcode reference library of European eukaryotic biodiversity, catalyzing species discovery and enabling comprehensive, harmonized species identification and biomonitoring, and supporting the global iBOL program. Immediate objectives include completing reference libraries for priority taxa, democratizing access to sequencing technologies, and strengthening a distributed community of practice. Key actions identified span five thematic areas: community building, sample collection and taxonomic verification, sequencing infrastructure, data management, and mainstreaming DNA-based approaches to meet societal needs. The strategy emphasizes integration with European research infrastructures to ensure long-term sustainability and resilience for biodiversity genomics in Europe.

Species discovery and specimen identification are of central importance in biology and in facilitating human interactions with nature. Assessing how many species exist, where they occur, and being able to assign unknown specimens and samples to known taxa underpins the conservation and utilization of the world’s biological resources. This fundamental societal need for telling species apart was the motivation for the development of DNA barcoding—the use of short, standardized regions of DNA as a universal tool for species discovery and species identification. The concept of DNA barcoding was first proposed by Hebert and colleagues (2003) for animals and has now been applied to all major branches of eukaryotic life (Hebert et al. 2016a, Gostel and Kress 2022). Importantly, DNA barcodes provide a cost-effective, high-throughput, scalable method of species discrimination and species identification (Hebert et al. 2025). Where species-level resolution is not achieved, the standard barcoding regions can be augmented to increase resolution (Coissac et al. 2016). More than 20 years since the original paper proposing DNA barcoding (Hebert et al. 2003), the method is now widely used around the world and represents an important tool to address the global taxonomic impediment (Ramsay 1986, Meier et al. 2025).

From its inception, the international DNA barcoding community recognized the need for global collaboration, and the community coalesced around common challenges, standards, and ways of working. This resulted in the launch of the International Barcode of Life (iBOL) consortium in 2007 (Hebert et al. 2016a). The first formal project undertaken by iBOL was named “Barcode 500K” and aimed to establish protocols, processes, and collaborations to generate DNA barcodes for 500,000 species. Following its successful completion, the second major project of iBOL, “BIOSCAN”, was launched in 2019, with three primary aims: (1) species discovery—focusing on an extended drive toward building the DNA barcode library and completing the inventory of life on earth, with the goal of DNA barcoding 10 million specimens; (2) species interactions—providing new insights into ecological processes by DNA barcoding suites of interacting species—the symbiome—including parasites, parasitoids, commensals, pathogens, pollinators, and prey species; and (3) biomonitoring baselines—establishing a network of DNA-based monitoring stations to lay the foundation for a future comprehensive DNA-based global biosurveillance system (Hobern 2021).

iBOL Europe, the European node of iBOL, was launched in 2021 to coordinate and further facilitate interactions and knowledge exchange across Europe. iBOL Europe was designed as a nexus for the DNA barcoding community in Europe, organizing meetings and fora for community connectivity and collaboration. Following its initiation, support from the Horizon Europe program via the Biodiversity Genomics Europe project (BGE) has enabled an expansion of activities and profile. Building on the growth of the DNA barcoding community in Europe, the purpose of this paper is to outline a joint mission and strategy for iBOL Europe. A strategy for iBOL Europe is necessary to guide activities of the network, to promote synergies, and to harness community support toward an agreed direction of travel within Europe that also complements and supports the wider goals of the global iBOL consortium.

The strategy for iBOL Europe presented here was first developed during a workshop at the University of Lodz, Poland, in June 2025, and is based on input from national representatives/leads of barcoding initiatives across 24 different European countries, as well as the iBOL leadership at the Centre for Biodiversity Genomics at the University of Guelph. In this paper, we first outline a high-level mission for iBOL Europe, then the immediate priority goals, which in turn are supported by a set of priority actions and activities for delivery. We expect this strategy to evolve and be refined with future input from the community.

## iBOL Europe mission

Our *mission* for iBOL Europe is to produce, curate, and provide access to a complete DNA barcode reference library of European eukaryotic biodiversity, catalyzing species discovery and enabling comprehensive, harmonized species identification and biomonitoring, and supporting the global iBOL program.

Delivery of this mission will require the establishment and long-term coordination of a distributed pan-European biodiversity genomics infrastructure, with strong representation of national and regional hubs. It will also require an inclusive program to build expertise and capacity, and promote close integration with the wider landscape of biodiversity science, genomics, environmental policy, nature conservation, and European research infrastructures (RIs).

## iBOL Europe immediate goals

Our *immediate* strategic goals (2026–2031) toward achieving the iBOL Europe mission are as follows:

- Completing and curating the DNA barcode reference library for species of known socioeconomic and conservation importance in Europe and supporting DNA-based biomonitoring to meet societal needs, prioritizing:
  - Endemic, rare, and endangered species, including species from biodiversity hotspots, threatened habitats, and/or underrepresented biogeographic regions;
  - Keystone species and taxa that underpin ecosystem functions and whose health is critical for the provision of ecosystem goods and services;
  - Indicator species and taxonomic groups relevant for environmental monitoring and ecological assessments;
  - Invasive and pest species whose detection and control are important for environmental health and management;
  - Common adulterants and contaminants of consumer products to support product authentication;
  - CITES/traded species where species identification supports regulatory oversight and enforcement;
  - Highly diverse, poorly understood species-rich groups to enable biodiversity characterization and the connection of DNA sequences with species traits and their potential roles within ecosystems;
  - Increasing phylogenetic coverage and representation to enhance understanding of European biodiversity and its functions.
- Strengthening and growing a distributed and connected community of practice by:
  - Democratizing access to sequencing technologies, computational tools, and analytical workflows;
  - Bridging the knowledge and capacity gaps across Europe to ensure equitable participation;
  - Providing training and capacity building to biodiversity researchers and end-users;
  - Upscaling the production of data and their translation into practical applications that benefit science and society according to the FAIR and CARE principles (Wilkinson et al. 2016, Caroll et al. 2020);
  - Developing and enhancing strategic partnerships with complementary organizations and initiatives to grow European and global capacity in biodiversity characterization, conservation, and utilization.

BOX 1. **Key initiatives relevant to iBOL Europe**

**Biodiversity genomics**

*Biodiversity Genomics Europe (BGE)*, a European consortium supported by Horizon Europe project funding, whose aim is to accelerate the use of genomic science to enhance understanding of biodiversity, monitor biodiversity change, and guide interventions to address its decline, bringing together the iBOL Europe and ERGA communities (www.biodiversitygenomics.eu/);

*International Barcode of Life (iBOL)*, a global initiative building a globally accessible DNA-based system for the discovery and identification of all multicellular life (www.ibol.org/);

*Earth Biogenome Project (EBP)*, a global project aiming to sequence, catalogue, and characterize the genomes of all of Earth’s eukaryotic biodiversity (www.earthbiogenome.org/);

*European Reference Genome Atlas (ERGA)*, the European node of the EBP, coordinating and delivering the production and use of reference genomes from European biodiversity (www.erga-biodiversity.eu/);

*International eDNA Standardization Task Force*, a global initiative focused on developing and implementing standardized methods for environmental DNA (eDNA) data collection and interpretation (www.iestf.global/);

*eDNA^Europe^*, an initiative exploring the potential for establishing a European society focusing on environmental DNA;

*Global Genome Biodiversity Network (GGBN)*, a global network of collections of genomic samples from across the Tree of Life (www.ggbn.org/).

**European infrastructures and partnerships**

*Biodiversa+*, a European biodiversity partnership supporting biodiversity research with an impact on policy and society (www.biodiversa.eu/);

*Consortium of European Taxonomic Facilities (CETAF)*, a European network of biological and geological collections focusing on taxonomy and systematic biology (www.cetaf.org/);

*Distributed System of Scientific Collections (DiSSCo)*, a new research infrastructure for natural science collections aiming to digitally unify all European natural science assets, sharing common access, curation, policies, and practices across countries (www.dissco.eu/);

*ELIXIR*, a European research infrastructure that coordinates and develops life science resources across Europe so that researchers can more easily find, analyze, and share data; exchange expertise; and implement best practices (www.elixir-europe.org/);

*EU Biodiversity Observation Coordination Centre (EBOCC)*, a planned permanent infrastructure to coordinate and foster the generation and use of high-quality data to underpin the biodiversity knowledge base used across EU policies (https://preprints.arphahub.com/article/128042/);

*Integrated European Long-Term Ecosystem Research (eLTER)*, an emerging Research Infrastructure whose aim is to facilitate high-impact research and catalyze new insights about the compounded impacts of climate change, biodiversity loss, soil degradation, pollution, and unsustainable resource use on a range of European ecosystems and socio-ecological systems (www.elter-ri.eu/).

**Other related global programs**

*Global Biodiversity Information Facility (GBiF)*, an international network and data infrastructure aimed at providing open access to data about all types of life on Earth (www.gbif.org/);

*Ocean Biodiversity Information Facility (OBIS)*, a global open-access data and information clearing-house on marine biodiversity for science, conservation, and sustainable development (www.obis.org/);

*Group on Earth Observations Biodiversity Observation Network (GEO BON)*, a global network of researchers dedicated to improving the acquisition, coordination, and delivery of biodiversity information at the global, regional, and national levels (www.geobon.org/).

## Priority actions and activities

**Figure 1.**
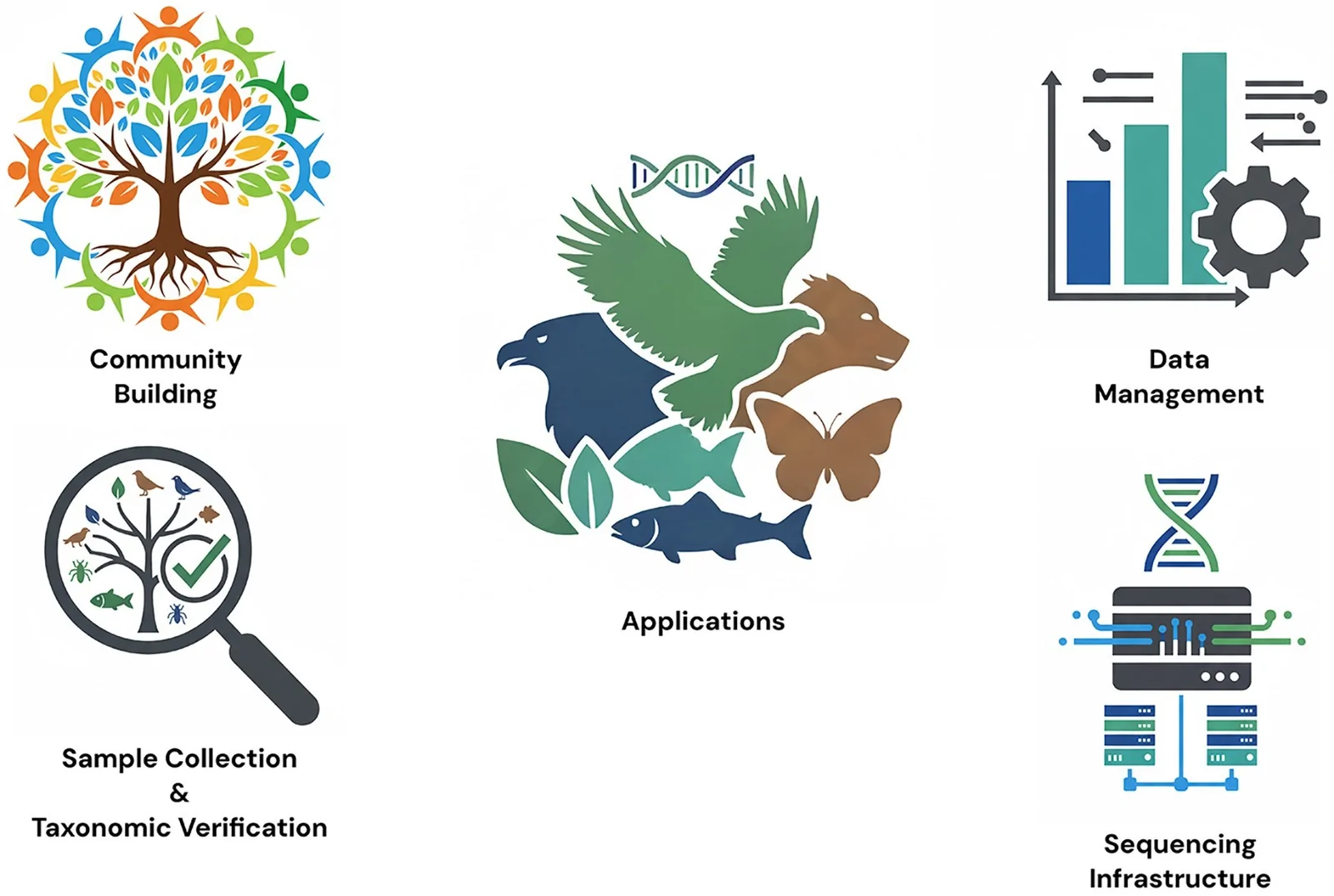
Thematic areas of priority actions and activities for iBOL Europe, figure remixed by Fantoni, K., using Gemini, 2025; CC-BY.

To enable the delivery of the iBOL Europe mission and to address immediate priorities, we have identified a set of priority actions and activities. These are structured across the following five thematic areas (figure 1) and discussed in turn below: (a) building the community, (b) sample collection, identification, and verification, (c) accessible and scalable sequencing, (d) data management and analysis, and (e) applications.

### Building the community

In early 2026, the iBOL Europe membership comprised 183 institutions and over 570 individual members from 45 countries, among which 14 are Inclusiveness Target Countries (ITCs).

The landscape of DNA barcoding activities in Europe includes a mixture of national programs, large-scale individual projects, smaller-scale projects and activities, and the activities of individual researchers (www.iboleurope.org/barcoding-landscape/). The landscape can also be split into well-funded DNA barcoding programs—mostly in Northern/Western Europe, and DNA barcoding programs in biodiversity-rich Southern and Eastern European countries that have access to less funding. This heterogeneity in levels and resourcing of activity has created a highly heterogeneous coverage of the DNA barcode reference library and deployment of DNA-based biomonitoring in Europe. Many of Europe’s biodiversity hotspots and centers of endemism remain vastly underrepresented. An additional component of this heterogeneity is uneven access to sequencing facilities and expertise across nations and regions, representing a significant barrier to progress. Differences in research culture and language barriers across countries represent additional challenges that can limit the process of community building and integration.

To support this heterogeneous and evolving network of practice and capacity, there is a need to support and nurture the European DNA barcoding community through inclusive knowledge exchange, resources, and information sharing. Strategic priorities include the following:

- *Developing National and Regional Nodes*, supporting the establishment and operations of barcoding networks within Europe, with a particular focus on national and regional barcoding initiatives for ITCs, and the establishment of a regional DNA-barcode node for Southern and Eastern Europe (box 2).
- *Understanding Community Needs*, continually assessing and monitoring the evolving needs of the DNA barcoding community, recognizing the diversity of contexts across Europe and globally.
- *Organizing Working Groups*, with a focus on identifying and tackling discrete areas of work necessary for meeting the goals and needs of the iBOL Europe community, including working groups focusing on training, reference library curation, and standards.
- *Fostering Collaboration and Engagement*, developing strategic partnerships with complementary initiatives (box 1), and supporting stakeholder engagement with diverse audiences, including policymakers, governmental agencies, nongovernmental organizations (NGOs), private companies, environmental, agricultural, forestry, fisheries, and public health sectors, and citizen scientists (c.f. Corrales et al. 2025).
- *Training and Capacity Building*, identifying gaps in knowledge and skills, and enabling equitable access and contribution to hands-on training, e-learning platforms, and other training materials to build skills and expertise leading to democratization of analytical capacity across regions. Example focal areas include train-the-trainer programs, and training courses on DNA barcoding using low-cost sequencing platforms, field collection strategies targeting priority taxa and geographic areas, and workshops or mentorships for writing funding proposals.
- *Communication and Outreach*, producing accessible, multilingual online resources (e.g., policy briefs), delivering science communication activities and public engagement events, maintaining communication platforms, and promoting outreach through social media. The activities should aim to improve communication within the DNA barcoding community in Europe, as well as wider international networking through the organization of scientific conferences and workshops.
- *Funding and Advocacy*, facilitating collaborative and strategic funding bids at various levels (national, regional, EU, and international), including funding bids to both private and public sectors.
- *Taxonomic Connections*, supporting continued integration of the DNA barcoding and taxonomic communities, including professional and amateur expert taxonomists/community scientists, to increase the quality of DNA barcoding reference libraries, as well as to bolster the recognition of taxonomic knowledge and expertise.
- *Inclusivity and Equity*, implementing positive actions and monitoring progress toward increased inclusivity of representation and equitable access and contribution to knowledge and resources for the DNA barcoding community in Europe. This includes cultivating and supporting growth in a diverse community of skills and knowledge to prevent the extinction of field experience and distinct taxonomic expertise.

BOX 2. **Toward a regional barcoding node for Southern and Eastern Europe**

Southern and Eastern Europe harbor a disproportionate concentration of global species diversity, endemic species, and threatened taxa, making it a critical area for biodiversity conservation (Coll et al. 2010, Maiorano et al. 2013). This richness stems from its unique climatic diversity, complex geography, and role as a crucial biogeographical crossroads. Key biodiversity hotspots include the Mediterranean Basin, particularly the Tyrrhenian islands, the Balkan, Iberian, and Apennine peninsulas with ancient lacustrine basins (e.g., Lake Ohrid and Lake Skadar) and diverse cave fauna, as well as the Carpathian and the Caucasus mountain ranges. These areas served as major glacial refugia, contributing significantly to Europe’s biological heritage. Various DNA barcoding initiatives are underway, such as national efforts in Bulgaria (BgBOL—www.bgbol.org/en), Greece (GrBOL—www.grbol.auth.gr/), Poland (PoLBOL—www.polbol.uni.lodz.pl/), Portugal (InBIO Barcoding Initiative; Ferreira et al. 2024), and Slovakia (AquaBOL—www.aquabol.sk/home/), as well as transnational initiatives such as the Caucasus Barcode of Life (CaBOL—https://ggbc.eu/), and collaborations focusing on specific taxonomic groups like European Lepidoptera, Odonata, or fish. However, a clear need persists for increased capacity and concerted activity. Many species in the Mediterranean and Eastern European regions still lack comprehensive reference coverage in global DNA barcode databases (Csabai et al. 2023), hindering effective species identification and timely conservation responses. Thus, a strategic priority for iBOL Europe, aligned with the broader iBOL mission to create a globally accessible DNA-based identification system for all multicellular life, is the establishment of a dedicated regional barcoding node for Southern and Eastern Europe. Such a node would provide focused impetus, directly addressing existing gaps in infrastructure, fostering regional collaboration, offering specialized training, and accelerating data generation to ensure the characterization, conservation, and sustainable utilization of the region’s biodiversity.

### Sample collection, identification, and taxonomic verification

In the DNA barcoding workflow, specimens must first be sampled in the field or chosen from existing collections, be identified, have appropriate metadata recorded, and (where not already available) be vouchered and digitized. Vouchering is a key step in the sampling process, and this preparation of physical museum specimens and/or imaging step is time-consuming, expensive, and often overlooked in funding proposals. Another key bottleneck for DNA barcoding remains the scarcity of taxonomic expertise for specimen identification and verification, set against the challenge of large numbers of undescribed or difficult-to-identify species, even in comparatively well-studied countries. This shortage of taxonomists, “the taxonomic impediment” (Ramsay 1986, Engel et al. 2021), thus necessitates (a) maximally efficient approaches for enhancing the connection and integration of taxonomic specialists into DNA barcoding programs, and (b) careful development of workflows that enable progress in megadiverse groups, in the absence of adequate available taxonomic expertise (Hebert et al. 2016b, Hartop et al. 2024). Strategic priorities include the following:

- *Sampling of Museum Specimens*, capitalizing on the globally important biological resources housed in Europe’s natural history collections, and acting as advocates for responsible tissue sampling, digitization, and metadata processing; seeking funds to support museum specimen identification and sampling (including type specimens); and promoting the use of shared protocols and standard operating procedures (SOPs) for museomics (box 3).
- *Organizing Targeted Field Sampling*, harnessing the networks of taxonomic experts (including amateur expert taxonomists, community scientists, and other citizen scientists) at international, national, and regional levels, arranging collecting and sample identification activities, and the consistent links of samples to voucher specimens and suitable metadata.
- *Deploying Sample Collection Devices*, supporting community activities to provide a source of material for biomonitoring (e.g., for bulk sample sequencing via metabarcoding) and/or dark taxa studies (sequencing-based characterization of poorly known megadiverse groups—megabarcoding, e.g., Hartop et al. 2024), and continually assessing the sustainability of sampling methods and approaches.
- *Maintaining a Dynamic European DNA Barcode Gap Analysis*, providing strategic oversight and regularly updated online resources for targeting and prioritizing sampling efforts to fill gaps across taxonomic groups, geographic regions, and habitat types.
- *Curating the European DNA Barcode Reference Library*, coordinating and facilitating community efforts to verify legacy and new barcode records to create high-quality curated reference resources that meet metadata standards and have reliable identifications that align with up-to-date taxonomies.
- *Enhancing AI-based Tools*, developing and integrating AI-based sorting, digitizing, curating, and phenotyping approaches into DNA barcoding workflows, enabling rapid and consistent selection of samples for sequencing, semi-automated curation processes, and the characterization and extraction of traits from sequenced specimens (Valan et al. 2019, Wührl et al. 2022).
- *Providing Guidance and Support for Sampling Permissions and Shipping*, facilitating smooth, legally compliant, and ethical sampling and transport of fresh and museum material for DNA barcoding.

BOX 3. **The value of European museums as a specimen source for DNA barcoding and biomonitoring**

European natural history collections host hundreds of millions of biological specimens, representing a very large proportion of all known species and many species yet to be formally described. These specimens are now amenable to large-scale DNA sequencing due to the reduced costs of short-read sequencing and recent developments in the field of museomics (Ferrari et al. 2023, Davis and Knapp 2025, Marsh et al. 2025).

This resource is of enormous value for DNA barcoding European and wider global biodiversity, as it contains large numbers of expertly identified and vouchered specimens, which can be accessed more efficiently than novel field collections. Furthermore, DNA sequencing of type specimens creates a clear link between taxonomic names and DNA sequence data, which is of great importance for building barcode reference libraries, connecting DNA sequence data with existing taxonomic and organismal knowledge (Ferrari et al. 2023, Letsch et al. 2025). Large-scale DNA barcoding of European museum specimens has, thus, the potential to make a major contribution to addressing the taxonomic impediment, and the benefits arising from sequencing these European museum collections will extend well beyond Europe, given the global coverage of the specimens.

Continued and extended dialogue and collaboration between genomic scientists and collection curators is key to ensuring mutual understanding and undertaking effective and sustainable sampling of museum collections for DNA sequencing, ensuring maximum value of museum material without compromising their future use (Ferrari et al. 2023). Likewise, clear understanding of any permit and wider constraints on museum specimen sequencing is essential to ensure effective legal and ethical use (Ferrari et al. 2023).

### Accessible and scalable sequencing

The global DNA barcoding community has benefited enormously from the sequencing facility at the Centre for Biodiversity Genomics at the University of Guelph, Canada. However, there is a clear need to provide complementary and additional strategic capacity to further develop DNA barcode data generation in Europe, and R&D to advance biodiversity monitoring. Our strategy for DNA sequencing for iBOL Europe recognizes the need for a multifaceted approach, including high-throughput sequencing centers with efficient robotics and the ability to process millions of specimens; dedicated museomic facilities for sequencing the historical specimens (including type specimens) held in European museums; and distributed low-cost sequencing facilities enabling an inclusive and geographically widespread community to engage with DNA barcode reference library construction. Strategic priorities include the following:

- *Community Support for Expansion of Existing Sequencing Facilities* enabling the delivery of DNA barcode reference sequences using high-throughput processing of heterogeneous tissue types, with protocols in place to accommodate a phylogenetically wide range of taxa.
- *Establishment of Distributed R&D Networks*, with investment and commitment to develop and share protocols for challenging samples, including historical specimens, and wider genomic approaches to generate DNA barcode reference sequences such as genome skimming (Coissac et al. 2016).
- *Museomics*, building and growing the science, culture, and practice of collection-sequencing in Europe, unlocking the vast resources of European natural science collections, via close collaboration with collection curators and the development and implementation of effective SOPs and protocols that maximize sequence recovery and minimize contamination risks and impacts on specimens (box 3).
- *Supporting the development of low-cost distributed sequencing facilities* to generate DNA barcode references, capitalizing on the availability of low-cost sequencing platforms and thus enabling a wider range of organizations to participate in data generation (box 4).
- *R&D on metabarcoding and eDNA approaches*, including development and calibration of protocols to underpin harmonization of methods across laboratories and institutions.
- *Assessment of Requirements, and Development of Services*, supporting high-throughput sample processing for DNA barcoding (beyond DNA sequencing), including robotic services for DNA extraction and/or PCR, to remove rate-limiting steps in reference library construction.

BOX 4. **Opportunities for low-cost DNA barcode sequencing in Europe**

The advent of third-generation sequencing technologies, in particular the Oxford Nanopore Technology (ONT) MinION device, enables low-cost, distributed, medium- to high-throughput DNA sequencing with comparatively little capital investment (Srivathsan et al. 2021, Hebert et al. 2025). The very small size and high portability of these sequencing machines mean that the equipment to set up a DNA barcoding facility does not have large spatial requirements and can, in principle, be set up at any location with access to electricity. Depending on the scale of multiplexing, the per-sample sequencing costs can be as little as €0.01, and bioinformatic tools to filter and quality assure raw sequencing data are now readily available (Hebert et al. 2025).

### Data management and data analysis

The baseline requirement for DNA barcoding data management and data analysis is the long-term sustainable provision of effective, robust, secure, and resilient workbenches and databases that support all major taxonomic groups, encompassing reference libraries as well as metabarcoding and eDNA applications. The delivery of such infrastructure will need to address multiple challenges, including (a) ensuring that DNA barcode databases and analytical tools evolve to meet the needs of DNA barcoding beyond existing markers and can accommodate new data types, data applications, and sequencing platforms; (b) providing interoperability and data exchange between different data systems and repositories; (c) satisfying European requirements for data sovereignty; (d) ensuring resilience and long-term support for key data assets and tools; and (e) being designed to allow development and augmentation by the wider community to harness distributed efforts and resources. Strategic priorities include the following:

- *European Support for Hosting and Development of the Barcode of Life Datasystem (BOLD)*, supporting the global initiative; adding system resilience and European sovereignty; its capacity for bulk data ingestion and hosting, data management, validation, and analysis; and supporting its long-term governance and management.
- *Development of DNA Barcode Systems, Tools, and Workflows*, extracting standard, alternative, and new, extended genomic DNA barcodes from genome skims and whole genomes; and utilizing the wider data that are produced (including organelle genomes and fractional components of the nuclear genome) for enhanced species identification and delineation.
- *Supporting and Contributing to Existing Community (meta)Data Standards*, embedding standards such as DarwinCore (DwC), Minimum Information about a Digital Specimen (MIDS), and Open Digital Specimen (OpenDS) throughout the European DNA barcoding data landscape, as well as implementing actionable persistent identifiers (PIDs) for all data types supporting the development of a fully FAIR-compliant data infrastructure.
- *Enabling Programmatic and Semantically Enabled Linkages between BOLD and Other Relevant Data Repositories*, supporting integrated analyses and complementarity of systems, including linkages between DNA barcode data, distributional data, other genomic data, taxonomy, and voucher-derived data (e.g., the developing DiSSCo IR of digitized European natural science collections).
- *Developing the Infrastructure for Gap Lists*, enabling the easy production of dynamic national-level, regional-level, and continental-level library barcode gap lists; identifying important gaps in reference libraries; and thus promoting complementarity and focus in collecting and sequencing activities.
- *Developing Best Practices for Data Management for Field Sampling*, enabling integration of DNA barcode gap lists with local checklists and supporting easy collection, taxonomic anchoring, and upload of sample metadata and images.
- *Supporting DNA Barcode Reference Library Curation*, building tools for library clean-up and curation, including taxonomists’ validation, resolution of taxonomic discordance, species discovery, and the creation of geographically and/or taxonomically focused reference libraries to support biomonitoring.

BOX 5. **The European barcoding data landscape**

The Barcode of Life Data System—BOLD (Ratnasingham et al. 2024) is the barcoding community’s preferred platform for barcoding data submission, validation, and publication. BOLD data records consist of the intersection between a DNA sequence, information on the specimen from which it was obtained (e.g., holding institution, taxonomic identifier, images), the collection event of the specimen (e.g., collector, geographic coordinates, date and time), and taxonomic information (e.g., identifier). BOLD also has functionality for managing checklists and creating data sets (reference libraries) with globally unique identifiers that can be referenced elsewhere. The RESL (Refined Single Linkage) algorithm within BOLD allows clustering of sequences from animal specimens into presumptive species, which are then allocated a BIN (Barcode Index Number) number as a proxy for species (especially useful for taxonomic groups where conventional Linnean taxonomy is far from complete; Ratnasingham and Hebert 2013).

Currently, in its fifth iteration, BOLD is moving toward a decentralized infrastructure that decomposes its previously integrated components for submission and publication into separate systems that can be deployed independently in multiple hosting locations. Chiefly developed by the Centre for Biodiversity Genomics (Guelph, Canada), the infrastructure is complemented by mBRAVE (Ratnasingham 2019), a data management and analysis platform for metabarcoding and eDNA analysis. Reference libraries created in BOLD can be referenced and used directly in mBRAVE for species identification from metabarcoding assays.

UNITE (Kõljalg et al. 2005) is a web-based database and resource focused on the nuclear ribosomal ITS region. The UNITE database is particularly important for the fungal community, and UNITE clusters ITS sequences to approximate the species level and publishes each species hypothesis with a DOI. The UNITE team, based in Tartu, Estonia, also develops the underlying database engine PlutoF (Abarenkov et al. 2010), which provides a workbench for field sampling and downstream data management for metabarcoding (e.g., of soil samples).

There are numerous related systems of considerable importance to the DNA barcoding community in Europe. Key among these are the DNA sequence databases under the International Nucleotide Sequence Database Consortium (INSDC, e.g., GenBank and the European Nucleotide Archive), from which BOLD and UNITE obtain some of their data; the Distributed System of Scientific Collections (DiSSCo), which enriches and interlinks European specimen data; the Global Biodiversity Information Facility (GBIF) and the Ocean Biodiversity Information Facility (OBIS), which aggregate specimen and collection events; and the Catalogue of Life (CoL), the World Register of Marine Species (WORMS), and the World Flora Online (WFO), which aggregate authoritative registries of species names.

### Applications

**Figure 2.**
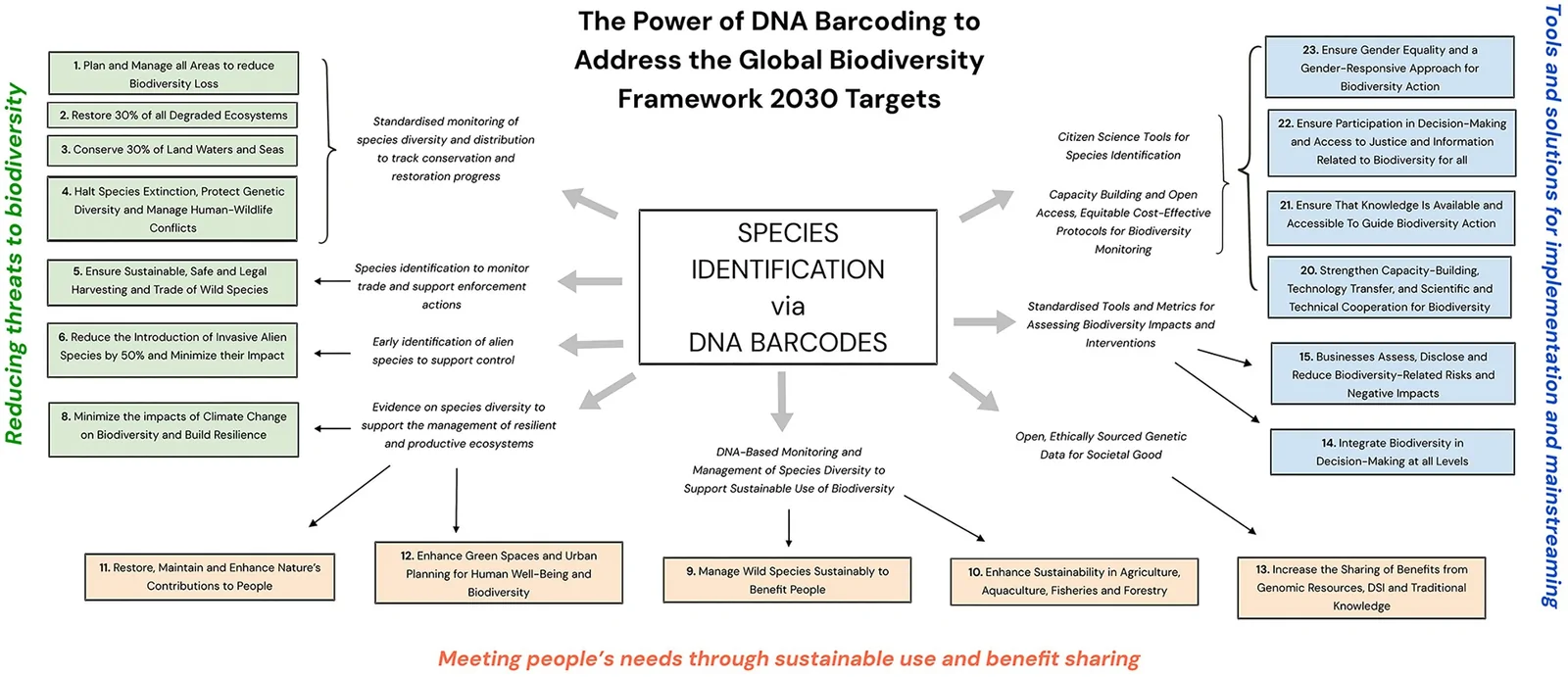
The power of DNA Barcoding to Address the Global Biodiversity Framework 2030 Targets, 2025, CC-BY-SA.
- *Connectivity to Key eDNA-based Biomonitoring Initiatives*, ensuring synergy between the iBOL Europe community and related initiatives, including the nascent European eDNA Society and the International eDNA Standards Task Force (box 1).
- *Supporting DNA-based Biomonitoring Deployment*, facilitating the routine use of DNA-based identification and monitoring across a wide spectrum of user needs and applications, including environmental impact assessments, biomonitoring of biodiversity hotspots, and the conservation management of protected areas and species.
- *Supporting Applications of DNA Barcoding to Support the Bioeconomy, Forensics, and Wider Commercial Services*, enabling routine deployment of DNA barcode methods for applied biomonitoring, commercial, and legal service provision, including the development of business models that support financial resilience and reinvesting of funds to support DNA barcode reference library production.

DNA barcode data support a wide range of applications, including taxonomy and systematics, DNA-based biomonitoring, product authentication, human and wildlife forensics, and the study of ecological interactions and dynamics. Underpinning this range of applications are (a) the need for comprehensive, reliable, and accessible DNA barcode reference libraries; (b) the need for clear guidance on the interpretation of metabarcoding and eDNA assays; and (c) appropriate awareness and understanding of DNA barcoding in the stakeholder community to engender uptake, interpretation, use, and mainstreaming. Strategic priorities include the following:

- *Strengthening Stakeholder Engagement*, ensuring the needs of the different stakeholder communities are understood, and providing guidance and roadmaps to support the mainstreaming of DNA-based species identification for various applications.
- *Advancing the Integration of DNA-based Methods into Policy and Practice*, demonstrating the role of DNA barcoding in achieving goals and targets under major national, regional, European, and global policy initiatives, such as the Global Biodiversity Framework (figure 2).

BOX 6. **Practical applications**

DNA barcoding has been widely used in Europe to address practical challenges in the assessment, management, conservation, and utilization of biodiversity (Laamanen et al. 2025), and is well aligned to meet the targets of the Kunming–Montreal Global Biodiversity Framework (figure 2). Examples include:

*Undertaking large-scale monitoring of insect diversity to better understand insect declines and the associated consequences*. Buchner and colleagues (2025) used DNA barcoding of Malaise traps to identify >30,000 species of insects in Germany, demonstrating a cost-effective blueprint for monitoring insects in near real time. Likewise, the Norwegian National Insect Monitoring Program has been running since 2020 with an annual increase in geographic coverage, currently using metabarcoding to analyse more than 1000 Malaise trap samples from 250 localities per year, and has detected more than 27,000 species since its start (Åström et al. 2025).

*Detecting rare and/or difficult to survey species using traces of DNA they shed into the environment*. Ballini and colleagues (2024), for instance, developed an assay to assess the presence of two endangered freshwater species, the white-clawed crayfish *Austropotamobius pallipes*, and Eurasian otter *Lutra lutra*, across four river catchments in Italy, and successfully detected the Eurasian otter DNA in rivers where the species was not recorded via field observations. Likewise, Leidenberger and colleagues (2025) created a barcode reference library and applied this in metabarcoding to detect rare species of mollusks in freshwater lakes and rivers in Sweden as part of a broader biodiversity assessment of water quality.

*Designing and monitoring the outcomes of conserving translocations*. Seymour and colleagues (2025) used eDNA to inform translocation efforts for the conservation of European green toad populations in Sweden, identifying favorable locations from metabarcoding-derived signatures of species positively associated with green toad occurrence.

*Detection and identification of invasive species to enable control and management interventions*. Eroğlu and colleagues (2025) used eDNA analyses to detect the presence of non-native fish species in Turkish rivers; likewise, Frigerio and colleagues (2024) used DNA barcoding for the early detection of invasive plant species in urban and peri-urban areas. Zangaro and colleagues (2024) used standard barcoding to identify and eDNA metabarcoding to screen for the nonindigenous decapod species *Penaeus aztecus* in the Aquatina di Frigole coastal lagoon in Italy, demonstrating the role of barcoding to screen for invasive species in this NATURA 2000 protected ecosystem and other Mediterranean protected areas.

*Assessing habitat quality and ecological health via DNA-based assessment of species assemblages*. Macher and colleagues (2025) showed how DNA barcoding of benthic invertebrates could be used to assess the ecological state of sites, supporting monitoring and reporting under the Water Framework Directive.

*Undertaking product authentication, using DNA barcoding to support consumer confidence*. Examples include authentication tests/biological identification of traded products, ranging from herbal supplements (Ruhsam and Hollingsworth 2018) to seafood (Barendse et al. 2019), and assessments of whether samples in trade belong to endangered species (Goymer et al. 2023).

Above and beyond these individual applications, there is now a growing body of countries developing national roadmaps and plans for implementing applied DNA-based biomonitoring and other applications, with a particularly influential example being the national roadmap for eDNA and other DNA-based methods in Finland (Norros et al. 2022).

## Concluding remarks

This outline of strategic priorities for iBOL Europe has identified five key priority actions that can be summarized as, firstly, developing and supporting a pan-European inclusive community of practice, building capacity, and sharing skills and expertise. Secondly, supporting the collection and identification of biological samples and curation of DNA barcode reference libraries, with a particular focus on strengthening integration between the DNA barcoding community and taxonomists, and capitalizing on the extensive natural science collections held in European museums and herbaria. Thirdly, facilitating the development of a distributed DNA sequencing infrastructure for DNA barcoding, including high-throughput sequencing centers, museomics laboratories, and distributed low-cost sequencing facilities. Fourthly, supporting and developing a robust, resilient, and connected data-management and data-analysis landscape in Europe for DNA barcoding, which evolves to meet diverse and changing community needs. And fifthly, enabling progress toward mainstreaming DNA-barcoding approaches for biodiversity characterization, biomonitoring, and applied species identification.

Although many of these ambitious strategic objectives can be advanced through coordinated community efforts, grant funding, and alignment with existing programs, the full realization of DNA barcoding’s potential at a pan-European scale requires sustained, long-term investment. Reliance on short-term project-funding risks perpetuating cycles of discontinuity, leading to loss of expertise, fragmentation of networks, and vulnerability in laboratory and data infrastructures. To mitigate these risks, we advocate exploring longer-term opportunities for integrating DNA barcoding into the European landscape of RIs. European RIs provide resources and services for research and innovation (European Commission: Directorate-General for Research and Innovation 2025). Embedding DNA barcoding into this framework would provide the stability, scalability, and resilience needed to support enduring progress and ensure that species identification and biomonitoring become permanent, well-supported pillars of European science and policy, enabling robust responses to biodiversity loss and environmental change.

The strategic priorities outlined here were developed for Europe, and reflect Europe’s biodiversity and the associated policy and application landscape, along with Europe’s national, regional, and continental scientific capacity and capabilities. However, these priorities have broader global relevance. Firstly, European efforts on method development and supporting the DNA barcoding data infrastructure (e.g., boxes 4 and 5) provide wider international benefits and augment the major contribution to date from the Centre for Biodiversity Genomics at the University of Guelph, Canada, in building global capacity and leading the global iBOL program. Secondly, DNA barcoding of European museum specimens (box 3), including those collected outside of Europe, will make a major contribution to democratizing access to the information held in these important specimens and supporting biodiversity characterization in the regions they were originally gathered from. Thirdly, the model of collaboration developed here among different countries representing heterogeneous cultures, political contexts, and differences in resource availability has potential for export to other continental or regional settings. A clear challenge for global biodiversity characterization is achieving synergies and complementarity of efforts, maximizing the efficiency of resource allocation, and coordinating efforts across neighboring countries. Our strategy for the International Barcode of Life in Europe provides a template and identifies key areas of work, some of which may be of value for the development of parallel strategies in other parts of the world.

## Data Availability

No data were generated or analyzed in support of this article.

## References

[bib1] Abarenkov K , et al. 2010. PlutoF—A web based workbench for ecological and taxonomic research, with an online implementation for fungal ITS sequences. Evolutionary Bioinformatics Online. 6: 189–196.

[bib2] Åström J , et al. 2025. Norsk insektovervåking. Rapport fra feltsesongen 2024. NINA Rapport 2526. Norsk institutt for naturforskning. (http://hdl.handle.net/11250/3167288).

[bib3] Ballini L, Ottonello D, Repetto V, Natali C, Chini G, Tolve L, Ciofi C, Fratini S, Iannucci A. 2024. Early detection of rare and elusive endangered species using environmental DNA: A case study for the Eurasian otter and the white-clawed crayfish in northwestern Italy. Conservation Genetics. 25: 999–1005.

[bib4] Barendse J, Roel A, Longo C, Andriessen L, Webster LMI, Ogden R, Neat F. 2019. DNA barcoding validates species labelling of certified seafood. Current Biology. 29: R198–R199. 30889387 10.1016/j.cub.2019.02.014

[bib5] Buchner D , et al. 2025. Upscaling biodiversity monitoring: Metabarcoding estimates 31,846 insect species from Malaise traps across Germany. Molecular Ecology Resources. 25: e14023. 39364584 10.1111/1755-0998.14023 PMC11646302

[bib6] Carroll S , et al. 2020. The CARE Principles for indigenous data governance. Data Science Journal. 19: 1–12.

[bib7] Coissac E, Hollingsworth PM, Lavergne S, Taberlet P. 2016. From barcodes to genomes: Extending the concept of DNA barcoding. Molecular Ecology. 25: 1423–1428. 26821259 10.1111/mec.13549

[bib8] Coll M , et al. 2010. The biodiversity of the Mediterranean Sea: Estimates, patterns, and threats. PLoS ONE. 5: e11842. 20689844 10.1371/journal.pone.0011842 PMC2914016

[bib9] Corrales C, Bacela-Spychalska K, Buzan E, Ekrem T, Ferreira S, Goodall-Copestake W, van Ommen Kloeke E, Hollingsworth PM, Bourlat SJ. 2025. The role of community science in DNA-based biodiversity monitoring. Molecular Ecology. 34: e70100. 40937587 10.1111/mec.70100 PMC12456123

[bib10] Csabai Z, Čiamporová-Zaťovičová Z, Boda P, Čiampor F. 2023. 50%, not great, not terrible: Pan-European gap-analysis shows the real status of the DNA barcode reference libraries in two aquatic invertebrate groups and points the way ahead. Science of the Total Environment. 863: 160922. 36539085 10.1016/j.scitotenv.2022.160922

[bib11] Davis CC, Knapp S. 2025. Exploring biodiversity through museomics. Nature Reviews Genetics. 26: 149–150. 10.1038/s41576-024-00801-2 39506143

[bib12] Engel MS , et al. 2021. The taxonomic impediment: A shortage of taxonomists, not the lack of technical approaches. Zoological Journal of the Linnean Society. 193: 381–387.

[bib13] Eroğlu M, Çelik I, Düşükcan M, Ünal EM, Çoban MZ, Gündüz F, Keskin E. 2025. DNA barcoding of invasive *Gambusia holbrooki* Girard, 1859 and *Atherina boyeri* Risso, 1810 inhabiting Upper Euphrates River Basin, Türkiye. Scientific Reports. 15: 1907. 39809825 10.1038/s41598-025-85828-z PMC11733162

[bib14] European Commission: Directorate-General for Research and Innovation . 2025. European Strategy on Research and Technology Infrastructures. Publications Office of the European Union. (https://data.europa.eu/doi/10.2777/9553939)

[bib15] Ferrari G , et al. 2023. Developing the protocol infrastructure for DNA sequencing natural history collections. Biodiversity Data Journal. 11: e102317. 38327316 10.3897/BDJ.11.e102317 PMC10848826

[bib16] Ferreira S , et al. 2024. The InBIO Barcoding Initiative Database: DNA barcodes of Portuguese moths. Biodiversity Data Journal. 12: e117169. 38903959 10.3897/BDJ.12.e117169 PMC11188589

[bib17] Frigerio J, Ouled Larbi M, Guidi Nissim W, Grassi F, Cortis P, Labra M. 2024. Early molecular detection of invasive alien plants in urban and peri-urban areas. Diversity. 16: 647.

[bib18] Gostel MR, Kress WJ. 2022. The expanding role of DNA barcodes: Indispensable tools for ecology, evolution, and conservation. Diversity. 14: 213.

[bib19] Goymer A, Steele K, Jenkins F, Burgess G, Andrews L, Baumgartner N, Gubili C, Griffiths AM. 2023. For R-eel?! Investigating international sales of critically endangered species in freshwater eel products with DNA barcoding. Food Control. 150: 109752.

[bib20] Hartop E, Lee L, Srivathsan A, Jones M, Peña-Aguilera P, Ovaskainen O, Roslin T, Meier R. 2024. Resolving biology’s dark matter: Species richness, spatiotemporal distribution, and community composition of a dark taxon. BMC Biology. 22: 215. 39334308 10.1186/s12915-024-02010-z PMC11438253

[bib21] Hebert PDN, Cywinska A, Ball SL, deWaard JR. 2003. Biological identifications through DNA barcodes. Proceedings of the Royal Society of London. 270: 313–321. 10.1098/rspb.2002.2218 PMC1691236 12614582

[bib22] Hebert PDN, Floyd R, Jafarpour S, Prosser SWJ. 2025. Barcode 100 K specimens: In a single nanopore run. Molecular Ecology Resources. 25: e14028. 39387679 10.1111/1755-0998.14028 PMC11646297

[bib23] Hebert PDN, Hollingsworth PM, Hajibabaei M. 2016a. From writing to reading the encyclopedia of life. Philosophical Transactions of the Royal Society B: Biological Sciences. 371: 20150321. 10.1098/rstb.2015.0321 PMC4971178 27481778

[bib24] Hebert PDN, Ratnasingham S, Zakharov EV, Telfer AC, Levesque-Beaudin V, Milton MA, Pedersen S, Jannetta P, deWaard JR. 2016b. Counting animal species with DNA barcodes: Canadian insects. Philosophical Transactions of the Royal Society B: Biological Sciences. 371: 20150333. 10.1098/rstb.2015.0333 PMC4971185 27481785

[bib25] Hobern D. 2021. BIOSCAN: DNA barcoding to accelerate taxonomy and biogeography for conservation and sustainability. Genome. 64: 161–164. 32268069 10.1139/gen-2020-0009

[bib26] Kõljalg U , et al. 2005. UNITE: A database providing web-based methods for the molecular identification of ectomycorrhizal fungi. New Phytologist. 166: 1063–1068. 15869663 10.1111/j.1469-8137.2005.01376.x

[bib27] Laamanen T , et al. 2025. Technology readiness level of biodiversity monitoring with molecular methods—where are we on the road to routine implementation?. Metabarcoding and Metagenomics. 9: e130834.

[bib28] Leidenberger S, Wiese V, Schaumann F, Pleiss F, Langen K, Bourlat SJ. 2025. Freshwater mollusc community screening—Classical and eDNA monitoring methods to detect rare, indicator and invasive species. Science of the Total Environment. 958: 177763. 39644641 10.1016/j.scitotenv.2024.177763

[bib29] Letsch H , et al. 2025. Type Genomics: A framework for integrating genomic data into biodiversity and taxonomic research. Systematic Biology. 74: 1029–1044. 40392669 10.1093/sysbio/syaf040 PMC12712332

[bib30] Macher T-H , et al. 2025. Fit for purpose? Evaluating benthic invertebrate DNA metabarcoding for ecological status class assessment in streams under the Water Framework Directive. Water Research. 272: 122987. 39708381 10.1016/j.watres.2024.122987

[bib31] Maiorano L , et al. 2013. Threats from climate change to terrestrial vertebrate hotspots in Europe. PLoS ONE. 8: e74989. 24066162 10.1371/journal.pone.0074989 PMC3774810

[bib32] Marsh WA, Hall A, Barnes I, Price B. 2025. Facilitating high throughput collections-based genomics: A comparison of DNA extraction and library building methods. Scientific Reports. 15: 6013. 39972011 10.1038/s41598-025-88443-0 PMC11839992

[bib33] Meier R, Srivathsan A, Oliveira SS, Balbi MIPA, Ang Y, Yeo D, Kjærandsen J, DdS A. 2025. “Dark taxonomy”: A new protocol for overcoming the taxonomic impediments for dark taxa and broadening the taxon base for biodiversity assessment. Cladistics. 41: 223–238. 39956942 10.1111/cla.12609 PMC11891956

[bib34] Norros V , et al. 2022. Roadmap for Implementing Environmental DNA (eDNA) and Other Molecular Monitoring Methods in Finland—Vision and Action Plan for 2022–2025. Finnish Environment Institute. Report no. 20/2022.

[bib35] Ramsay GW. 1986. The taxonomic impediment to conservation. Weta. 9: 60–62.

[bib36] Ratnasingham S , et al. 2024. BOLD v4: A centralized bioinformatics platform for DNA-based biodiversity data. Pages 403–441. in DeSalle R, ed. DNA Barcoding: Methods and Protocols, vol. 2744. Springer US. 10.1007/978-1-0716-3581-0_26 38683334

[bib37] Ratnasingham S. 2019. mBRAVE: The multiplex barcode research and visualization environment. Biodiversity Information Science and Standards. 3: e37986.

[bib38] Ratnasingham S, Hebert PDN. 2013. A DNA-based registry for all animal species: The barcode index number (BIN) system. PLOS ONE. 8: e66213. 23861743 10.1371/journal.pone.0066213 PMC3704603

[bib39] Ruhsam M, Hollingsworth PM. 2018. Authentication of *Eleutherococcus* and *Rhodiola* herbal supplement products in the United Kingdom. Journal of Pharmaceutical and Biomedical Analysis. 149: 403–409. 29154110 10.1016/j.jpba.2017.11.025

[bib40] Seymour M , et al. 2025. Effective amphibian conservation monitoring and habitat assessment using eDNA. Environmental DNA. 7: e70192.

[bib41] Srivathsan A, Lee L, Katoh K, Hartop E, Kutty SN, Wong J, Yeo D, Meier R. 2021. ONTbarcoder and MinION barcodes aid biodiversity discovery and identification by everyone, for everyone. BMC Biology. 19:217. 34587965 10.1186/s12915-021-01141-x PMC8479912

[bib42] Valan M, Makonyi K, Maki A, Vondráček D, Ronquist F. 2019. Automated taxonomic identification of insects with expert-level accuracy using effective feature transfer from convolutional networks. Systematic Biology. 68: 876–895. 30825372 10.1093/sysbio/syz014 PMC6802574

[bib43] Wilkinson M , et al. 2016. The FAIR Guiding Principles for scientific data management and stewardship. Scientific Data. 3: 160018. 26978244 10.1038/sdata.2016.18 PMC4792175

[bib44] Wührl L, Pylatiuk C, Giersch M, Lapp F, von Rintelen T, Balke M, Schmidt S, Cerretti P, Meier R. 2022. DiversityScanner: Robotic handling of small invertebrates with machine learning methods. Molecular Ecology Resources. 22: 1626–1638. 34863029 10.1111/1755-0998.13567

[bib45] Zangaro F, Pinna M, Specchia V. 2024. Environmental DNA as early warning for alien species in Mediterranean coastal lagoons: Implications for conservation and management. Diversity. 16: 525.

